# THE IMPACT OF THE COVID-19 PANDEMIC ON TEMPORARY AGENCY CARE STAFF USE IN OREGON ASSISTED LIVING

**DOI:** 10.1093/geroni/igae098.0226

**Published:** 2024-12-31

**Authors:** Sarah Dys, Ozcan Tunalilar, Yuliana Levchenko

**Affiliations:** Portland State University, Portland, Oregon, United States; Portland State University, Portland, Oregon, United States; Portland State University, Portland, Oregon, United States

## Abstract

Temporary agency staff (TAS) use in long-term care has become a spotlight issue due to workforce shortages and increased dependence during the COVID-19 pandemic. Nursing homes (NH) and assisted living communities (AL) rely on TAS to maintain compliance with staffing standards and meet residents’ needs. Settings with high TAS utilization have been associated with more deficiency citations and lower quality ratings. While recent NH studies show a substantial increase in chronic use of TAS, no similar studies document ALs’ use and change of TAS. Using data from a representative sample of Oregon ALs in 2016 and 2023, we compare differences in and organizational (license type, size, Medicaid contracts, profit status, rurality) correlates of TAS use by estimating linear and logistic regression models. Share of ALs with any TAS use increased significantly from 13% in 2016 to 22% in 2023 (p=0.012). In 2016, there were no differences in the likelihood of ALs using any TAS, except for size. In 2023, ALs with Medicaid contracts and in rural areas were 46% and 44% less likely to report using any TAS, respectively. TAS mix used also differed between 2016 and 2023. TAS use decreased for registered nurses (53.8% vs. 25.9%; p=0.005) and increased for licensed practical nurses (0.8% vs. 10.2%; p=0.060) and certified nursing assistants (6.5% vs. 38.2%; p<.001), with no significant difference in personal care aides. Findings contribute to understanding how ALs utilize TAS and can potentially add to policy debates around more stringent regulations for NH and AL staffing.

